# Food Supplements to Mitigate Detrimental Effects of Pelvic Radiotherapy

**DOI:** 10.3390/microorganisms7040097

**Published:** 2019-04-03

**Authors:** Charlotte Segers, Mieke Verslegers, Sarah Baatout, Natalie Leys, Sarah Lebeer, Felice Mastroleo

**Affiliations:** 1 Interdisciplinary Biosciences Group, Belgian Nuclear Research Centre (SCK•CEN), Boeretang 200, 2400 Mol, Belgium; charlotte.segers@sckcen.be (C.S.); mieke.verslegers@sckcen.be (M.V.); sarah.baatout@sckcen.be (S.B.); natalie.leys@sckcen.be (N.L.); 2 Department of Bioscience Engineering, University of Antwerp, Groenenborgerlaan 171, 2020 Antwerp, Belgium; sarah.lebeer@uantwerpen.be; 3 Department of Biotechnology, University of Ghent, Coupure Links 653, 9000 Ghent, Belgium

**Keywords:** intestine, radiation, microbiome, probiotic, prebiotic, vitamin

## Abstract

Pelvic radiotherapy has been frequently reported to cause acute and late onset gastrointestinal (GI) toxicities associated with significant morbidity and mortality. Although the underlying mechanisms of pelvic radiation-induced GI toxicity are poorly understood, they are known to involve a complex interplay between all cell types comprising the intestinal wall. Furthermore, increasing evidence states that the human gut microbiome plays a role in the development of radiation-induced health damaging effects. Gut microbial dysbiosis leads to diarrhea and fatigue in half of the patients. As a result, reinforcement of the microbiome has become a hot topic in various medical disciplines. To counteract GI radiotoxicities, apart from traditional pharmacological compounds, adjuvant therapies are being developed including food supplements like vitamins, prebiotics, and probiotics. Despite the easy, cheap, safe, and feasible approach to protect patients against acute radiation-induced toxicity, clinical trials have yielded contradictory results. In this review, a detailed overview is given of the various clinical, intestinal manifestations after pelvic irradiation as well as the role of the gut microbiome herein. Furthermore, whilst discussing possible strategies to prevent these symptoms, food supplements are presented as auspicious, prophylactic, and therapeutic options to mitigate acute pelvic radiation-induced GI injury by exploring their molecular mechanisms of action.

## 1. Local Radiotherapy as Pelvic Cancer Treatment

Pelvic cancers are among the most frequently diagnosed cancers worldwide [1], and pelvic radiotherapy is often an essential part of the multimodal therapeutic approaches [2,3]. To comfort the patients during their treatment, conventional fractionation is favored. Fractions are limited to 1.8–2 Gy/day, administered during five consecutive days. A full course may take up to 7 weeks or longer, which results in 50 Gy cumulated dose on average. Although it is widely used, a majority of pelvic cancer survivors suffers from a wide diversity of complications that collectively have been termed pelvic radiation disease [4]. 

## 2. Gastrointestinal Complications Associated with Pelvic Radiotherapy

Gastrointestinal (GI) complications may develop as result of the inevitable exposure of healthy intestinal tissue with an increased risk for the terminal ileum, colon, or rectum [5]. As a result, patients undergoing pelvic radiotherapy suffer from a significantly impaired quality of life, which adds an extra burden on top of the cost of health care.

In general, radiation-induced GI toxicity is labelled as acute or chronic clinical manifestations, both having a different pathogenesis. Acute radiotoxicity symptoms (nausea, diarrhea, abdominal pain, and fatigue) develop within the course of treatment or a period up to 90 days thereafter [5,6]. Nausea typically occurs relatively early, whilst diarrhea and abdominal pain become problematic 2–3 weeks after the start of radiotherapy. Although early events are frequently reported, they are generally considered reversible. Nevertheless, these symptoms may affect the patient significantly so that treatment interruption or original treatment plan adaptations may be required, which compromise the efficacy of tumor control. 

Symptoms of chronic radiotoxicity (altered intestinal transit, malabsorption [7], and dysmotility, which may progress to intestinal obstruction, fistula formation or intestinal perforation) appear after a latency period of 90 days to many years post-irradiation. They are mainly mediated by complex chronic processes including vascular sclerosis and transmural fibrosis [8]. Delayed events are less commonly reported but also less likely to reverse. Of note, acute and chronic radiation toxicities are not independent events, underlined by the consequential late effect theory saying that late injury is more likely to develop when severe acute toxicity exists [9,10,11]. Overall, radiation-induced GI toxicity is a progressive condition with a substantial long-term morbidity and mortality. 

### 2.1. Mechanisms of Pelvic Radiotherapy-Induced Effects to the Healthy Intestine

Although radiation-induced damage to the healthy intestinal tissue is a dynamic and progressive process, it is artificially divided into mucosal breakdown and subsequently induced inflammation as reviewed by François A. et al. [12]. In the following sections, we provide an overview of the underlying mechanisms of pelvic radiotherapy and its activated processes, highlighting the actions most relevant to discuss interference possibilities by food supplements. 

#### 2.1.1. Pelvic Radiotherapy-Induced Breakdown of Mucosal Homeostasis

The healthy intestine is a self-renewing tissue every 3–5 days throughout life [13,14]. This high turnover rate is sustained by a sub-population of stem cells that reside at the crypt base [15]. The resulting daughter cells exit the crypt and move to the amplifying transit compartment where they become precursor or progenitor cells. Subsequently, the cells migrate along the crypt/villus axis and differentiate into three different cell lineages, i.e., epithelial bordering cells, goblet cells, and entero-endocrine cells. The fourth cell lineage of Paneth cells moves back into the crypts [16]. 

**Figure 1:**
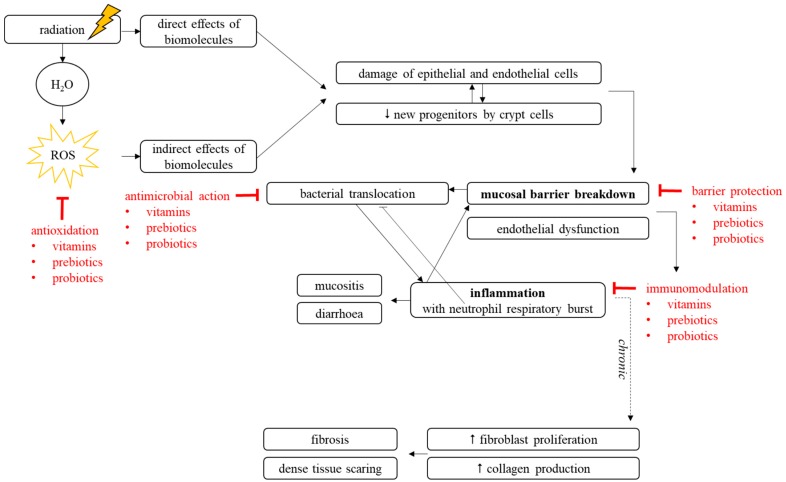
Scheme of radiotherapy inducing interactions between epithelial and endothelial radiation injuries in the gastrointestinal tract resulting in acute events and late tissue fibrosis.

Ionizing radiation mainly interacts with water molecules generating reactive oxygen species (ROS), which are considered as the most reactive molecules and thus capable of damaging DNA and other macromolecules (Figure 1) [17,18,19]. This may result in phenotypic modifications, alterations in downstream biological pathways, cell cycle arrest, and/or cell death by apoptosis, necrosis or mitotic catastrophe [19]. 

The sensitivity of any particular cell type to ionizing radiation is directly proportional to its mitotic rate and thus indirectly proportional to the extent of its differentiation [20]. Therefore, cells lining the GI tract are particularly vulnerable to the damaging effects of ionizing radiation, with stem cells being most radiosensitive. Within the intestine, two functionally distinct stem cells are distinguished, including Lgr5^+^ and Bmi1^+^ cells [21]. Lgr5^+^ stem cells are highly proliferating thereby maturing into epithelial cells whilst migrating towards the villus top [21,22]. These are highly sensitive to radiation and—within hours—quantitatively ablated by such procedures (Figure 1). The first two days following irradiation, an apoptotic phase, characterized by continuous crypt loss, shrinkage in crypt size, and shortening of the villi during this phase is apparent [23]. In contrast, Bmi1^+^ stem cells located at higher positions in the crypts are considered as a stem cell reservoir comprising less mitotically active and are thus more radioresistant cells [21]. Interestingly, Bmi1^+^ stem cells are activated two days after radiation-induced crypt apoptosis [23]. During this regeneration phase, Bmi1^+^ stem cells dramatically proliferate to repopulate intestinal crypts and villi [24]. The resulting, regenerated crypts are enlarged in size whilst the overall number of crypts decreases [25]. Eventually, during the normalization phase, the size of the crypts and the length of the villi are restored to pre-irradiation conditions [25].

After initial damage of Lgr5^+^ stem cells residing in the intestinal crypts, a tissue scaring process is initiated. Consequential late effects involve fibroblasts, which are stimulated upon exposure to radiation, which then enhance proliferation and collagen deposition, eventually resulting in tissue fibrosis [11]. Moreover, ionizing radiation is thought to induce a chronic, self-maintained scaring process driving fibrosis. However, during the acute phase, mucosal breakdown is induced through reduced expression of tight junctions and epithelial cell death compromising villous integrity, which leads to the development of inflammation, as described in the following section (Figure 1) [26]. 

#### 2.1.2. Pelvic Radiotherapy-Induced Inflammation

Ionizing radiation induces an inflammatory profile in healthy intestinal tissue. Due to disruption of the mucosal barrier, bacterial translocation is enabled, thereby triggering inflammation and immune cell recruitment (Figure 1) [12]. In addition, damaged vascular endothelium creates a proinflammatory, prothrombotic, and antifibrinolytic phenotype, which further stimulates increased secretion of cytokines, chemokines, and growth factors [27]. In parallel, the increased expression of adhesion molecules including VCAM-1, ICAM-1, PECAM-1, and selectins E and P are favored [28]. This proinflammatory state activates resident macrophages and enhances early extravasation of polymorphonuclear neutrophils (Figure 1) [29]. The presence of these neutrophils in tissues typically indicates an acute inflammatory profile, and is a hallmark of irradiated tissues [20,30]. Moreover, polymorphonuclear neutrophils exhibit microbicidal activity in case of bacterial translocation after vascular and mucosal breakdown [12]. ROS produced by the so-called neutrophils’ “respiratory burst” are crucial in the first control action of GI infection and inflammation. However, excessive and sustained ROS production damages neighboring healthy cells and tissues, and participates in the progression and the chronicity of radiation injury to the intestinal wall. The primary inflammatory state is then amplified through the additional recruitment and transmigration of monocytes, as well as the activation of resident mast cells (Figure 1) [12]. Both will stimulate the production of proinflammatory and profibrosing mediators such as IL-1β, IL-6, TGF-β, and TNFα (Figure 1) [12]. Importantly, the innate immune system recognizes motifs of bacteria, so called pathogen-associated molecular patterns (PAMPs), through pattern recognition receptors (PRRs) [31]. In addition, damage-associated molecular patterns (DAMPs) consisting of components of collectively dead/dying cells and damaged tissues can also activate PRRs. Among the different PRRs, toll-like receptors (TLRs) were the first family discovered and are best studied in their structure and activation mechanism. After exposure to ionizing radiation, TLRs present on the surface of multiple cell types including immune cells and intestinal epithelial cells (IEC) are reported to play an important role in tissue homeostasis and repair [32]. Of note, the gut microbiome was reported to influence the immune response development through blockage of specific immune checkpoints, which impacts cancer immunotherapy success [33]. The innate immune response characterized by macrophages, neutrophils, and mast cells is supported in a complementary way by the adaptive immune response regulated by B and T lymphocytes.

In general, the adaptive immune response is activated after a few days and necessitates the presentation of a specific antigen by specialized antigen-presenting cells such as dendritic cells (DCs) to B and T lymphocytes. As the rapid innate response develops within minutes after tissue irradiation exposure, micro-environmental changes favor the maturation of DCs. Antigen presentation then stimulates naive CD4^+^ T cells to differentiate into different T cell subsets such as Th1, Th2, Th17, or Treg cells [34]. Each of them shows a specific profile of cytokine expression assuming various redundant or opposed roles in tissue immune responses [35]. Overall, the continuing immune imbalance may play a significant role in the chronicity of radiation lesions [12]. 

In conclusion, the response of healthy intestinal tissue to radiation is mediated partly by cell death as well as activation of a strong oxidative and immune-modulated inflammatory component within all different tissue compartments (Figure 1). However, the precise roles of the different resident and attracted immune cells are still vague, as well as the contributions of the innate and adaptive immune responses. 

### 2.2. Pelvic Radiotherapy-Induced Effects on Gut Microbiota

Recently, microbiome research began to boom due to the development of 16S ribosomal RNA gene-sequence-based metagenetic methods resulting in major advantages in characterizing microbial populations [36]. The presence of 10^13^ bacterial cells in a healthy adult shows the high complexity and diversity of human microbiota [37]. Starting from the nose and mouth up until the rectum, microbiota differ in number and composition, whilst being highly susceptible to influences including the diet. Specifically, anaerobic bacteria represented by the genera *Bacteroides*, *Eubacterium*, *Bifidobacterium*, *Peptostreptococcus*, *Ruminococcus*, *Clostridium,* and *Propionibacterium* were characterized as the predominant (>10^9^ Colony Forming Units (CFU)/g)) microbiota of the human gut [38]. Multiple functions have been attributed to the gut microbiota including roles in the development of intestinal structure and morphology, metabolism like vitamin production and fermentation, neuroendocrine signaling through the gut-brain axis, as well as protection through immune system development and homeostasis [39,40]. Immune functions are generally based on the interaction of commensal bacteria with TLRs and subsequent NFκB signaling pathway activation. Furthermore, it has been suggested that microbiota have a crucial role in immune tolerance induction since germ-free animals lack this ability with regard to oral tolerance [41,42]. Despite incomplete knowledge regarding the underlying mechanisms, the mucosal microbiota are capable of limiting growth and even kill certain transient microbial pathogens that enter their habitat [43,44,45,46,47]. 

Changes in the gut microbial ecosystem are associated with dysbiosis, which can be defined in various ways, but generally refers to changes in microbial compositions that cause a drastic imbalance between beneficial and potentially pathogenic bacteria, which renders the gut more vulnerable to pathogenic insults [48]. Moreover, an imbalanced microbiome is characterized by changes in its functionality and metabolic activity, or changes in their local distributions along the GI tract. As a consequence, recent insights have linked gut microbiome dysbiosis with various disease states of which inflammatory bowel disease and cancer are well-known examples [49,50]. 

Radiation has also been defined as a stressor of the GI microbial ecosystem. Radiation-induced gut microbial dysbiosis was hypothesized to negatively contribute to pelvic radiation disease in cancer patients with associated mucositis, diarrhea, systemic inflammatory response, and fatigue [51,52]. This key role of gut microbiota in radiotherapy-induced GI toxicity has been known for decades. For instance, germ-free mice are known to be resistant to radiation-induced enteritis [53,54,55,56]. In addition, post-radiotherapy changes in gut microbial diversity have been described [57,58]. Additional clinical studies have shown that cancer patients exposed to radiation therapy exhibit marked alterations in gut microbiota composition [51,59,60]. Most frequently, a decrease in potentially protecting *Bifidobacterium*, *Clostridium* cluster XIVa, and *Faecalibacterium prausnitzii* were observed, together with an increase of *Enterobacteriaceae* and *Bacteroides*, which might contribute to the development of mucositis, and diarrhea in particular [60]. In addition, patients developing post-radiotherapy diarrhea compared to no diarrhea patients were characterized by an increase in *Actinobacteria* and *Bacilli*, as well as a decrease in *Clostridia* [57].

Notably, gut microbiota are not only affected by radiotherapy, but as importantly, the pre-exposure microbial profile is associated with the susceptibility of developing post-radiotherapy diarrhea [51]. For instance, a pilot study revealed that gut microbial diversity richness, and the *Firmicutes*/*Bacteroidetes* ratio were significantly altered prior to pelvic radiotherapy in patients who later developed diarrhea [51]. Unfortunately, the exact microbial biomarkers (specific taxa or biodiversity markers), which could predict this susceptibility have not yet been defined due to the highly individualized and fluctuating microbiome profiles, complex interactions between the host, clinical and environmental factors, as well as the heterogeneity of reported microbiome studies. In addition to biomarker discovery, increasing knowledge about important microbiome features will also allow the exploration of personalized radiotherapy delivery methods.

All this knowledge highlights the importance of re-establishing bacterial homeostasis after pelvic radiation, as well as prevention of dysbiosis induced by radiotherapy. One favorable way consists of the use of natural food supplements, being commonly used because of their long-standing positive association with health. These interventions will be discussed further in this review. 

## 3. Nutritional Interventions for Pelvic Radiotherapy-Induced Side Effects

To maintain a positive balance between the quality of tumor control by pelvic radiotherapy and the damage posed to healthy surrounding intestinal tissue, different strategies—technical and biological—have been developed. Here, we will focus on nutritional interventions including vitamins, prebiotics, and probiotics.

### 3.1. General Improvements in Gut Health

***Vitamins*** Vitamins are dietary micronutrients acting as cofactors for numerous enzymes essential for health. Among those, vitamins E, A, C, B6, B9, and B12 have been reported as potent GI radioprotectors, and will therefore be discussed here [61]. 

First, vitamin E is an essential nutrient for all animal species with many biological functions of which its antioxidative property is considered most important [62]. For instance, exogenous provision of vitamin E was reported to attenuate acute mountain sickness characterized by compromised intestinal integrity, with physical improvements of mountaineers at high altitudes [63,64,65]. 

Vitamin A derivatives are known for their pleiotropic effects on human health and to modulate several biological processes including intestinal immune and barrier functions [66,67,68]. Interestingly, supplementation has been shown to reduce infant mortality due to diarrhea in endemic areas of vitamin A deficiency [69]. 

Vitamin C (ascorbic acid) is broadly used as antioxidant and essential for normal cell function, growth and development and thus indispensable for human health and well-being [70,71]. For instance, vitamin C impacts wound healing processes through stimulation of collagen biosynthesis and early resolution of inflammation and tissue remodeling [72]. In view of this data, pre-operative vitamin C supplementation was shown to improve colorectal anastomotic healing and biochemical parameters in malnourished rats [73]. 

Finally, vitamin B6 (pyridoxine), B9 (folate), and B12 (cobalamin) are provided by food or retrieved from commensal bacteria that produce them in the gut. Generally, vitamin B6 is involved in various homeostatic processes in health and disease, including host immune responses [74]. Since their bioavailability is dependent on the correct function of the GI tract, vitamins B9/B12 deficiencies are associated with GI diseases including inflammatory bowel disease. Vitamin B9 supplementation on chemically-induced intestinal stress has yielded contradictory results [75,76].

***Prebiotics*** In 2017, the International Scientific Association of Probiotics and Prebiotics (ISAPP) updated the definition and scope of prebiotics to “a substrate that is selectively utilized by host microorganisms conferring a health benefit” [77].

Initially, oligosaccharides were considered as the main prebiotic source, with fructans (fructooligosaccharides (FOS) and inulin) and galactans (galactooligosaccharides (GOS)) in particular. Their effects on gut microbiota have been extensively investigated. For instance, preclinical studies uniformly reported changes in specific gut microorganisms of diet-induced obese and diabetic animals following prebiotic supplementation. Specifically, a decreased *Firmicutes*/*Bacteroidetes* ratio as well as the proportion of *Tenericutes*, *Cyanobacteria,* and *Verrucomicrobia* was observed. Moreover, this impacted several important metabolites implicated in metabolic disorders [78,79]. This effect was confirmed in obese women [80]. In addition to their metabolic influence, dietary FOS and GOS were reported to affect fecal microbiota by increasing *Bifidobacteria* abundance, which alleviated functional constipation in constipated subjects [81,82,83]. 

Another class of candidate prebiotics is polyphenolic phyco- and phytochemicals including anthocyanins, flavonoids, tannins, and lignins amongst others, which are highly enriched in cyanobacteria, plants, and herbs [84]. Most of them (95–99%) are not absorbed in the small intestine and reach the colon where they are subjected to microbial biotransformations producing bioactive metabolites [85]. Overall, these polyphenol-derived metabolites are possibly responsible for health benefits due to their ability to modulate intestinal bacterial populations [86]. This two-way interaction between an individual’s gut microbiota and polyphenols to determine health effects is well-established [87,88,89]. For instance, the prebiotic potential of cocoa flavanols was assessed by a cross-over intervention study in healthy volunteers, which reported increased *Bifidobacteria* and *Lactobacilli*, and decreased *Clostridia* counts [90]. Other studies reported the gut microbiota shaping potential of polyphenols and enriched food, which further promote health benefits related to inflammation and obesity [78,91,92,93]. 

***Probiotics*** Recently, ISAPP strengthened the FAO/WHO definition of probiotics: “live microorganisms that, when administered in adequate amounts, confer a health benefit on the host” [94]. The concept of probiotics is becoming increasingly popular, as evidenced by rapidly expanding research highlighting its beneficial effects, with lactic acid bacteria from the genera *Lactobacillus* and *Bifidobacterium* being the major representatives. General intestinal health effects including intestinal barrier improvements, inhibition of (opportunistic) pathogens or stimulation of beneficial microorganisms, and reduction of intestinal inflammation profiles were documented, in healthy and diseased animals and humans. A sample of in vitro and in vivo research is summarized in Table 1.

In addition to general intestinal health support, there is also convincing evidence supporting the efficacy of probiotics in the treatment of acute diarrhea, especially in children with rotavirus infection, likely attributed to the stimulation of IgA-specific antibody secreting cells [95,96]. Frequently studied probiotics in that field are *L. rhamnosus* GG and *Saccharomyces boulardii*. Guarino, Guandalini, and Lo Vecchio (2015) reviewed the modern perspective on the potential use of probiotic agents in the management of infectious diseases, with special emphasis on diarrheic symptoms [97].

### 3.2. Preclinical Evidence for Reduction of Radiation-Induced GI Toxicity

Since food supplements have been reported to improve general gut health as well as diarrheal diseases, it is of interest to investigate whether they can preserve surrounding, healthy tissues during radiotherapy and reduce radiation-induced GI toxicities. Both preclinical and clinical studies exploring their radioprotective properties are discussed in the following sections (summarized in Table 2 and Table 3).

***Vitamins*** Since ionizing radiation produces free radicals, antioxidative vitamins have been explored for their radioprotective potential [118]. For instance, dietary vitamin E (alpha tocopherol) pre-treatment preserved general small intestinal morphology of rodents against high doses of radiation [119,120,121]. Notably, the starting point of diet supplementation matters as lethality was only mitigated when antioxidant vitamin E supplementation was started 24 h after 8 Gy whole-body irradiation [122].

Pyridoxamine, one of the natural forms of vitamin B6, was shown to prevent apoptosis in small intestinal epithelium of mice exposed to 4 or 8 Gy whole body irradiation [123]. Preclinical, radioprotective effects of vitamins B9/B12 on the GI tract have not yet been reported.

Radioprotection by vitamin A is questionable. For instance, vitamin A had no effect on clinical parameters and could not protect mice against 7–10 Gy whole body irradiation-induced changes in intestinal sugar transport [124]. However, a combined cocktail of antioxidant vitamins A, C, and E before irradiation protected the absorptive function of the small intestine when tested in an identical experimental setup by the same research group [125]. 

Improved survival was reported with oral vitamin C pre-treatment, either by preventing small intestinal apoptosis in mice exposed to 14 Gy whole-body irradiation [126] or by reducing inflammatory cytokines and free radical metabolites after 7–8 Gy whole-body irradiation of mice [127]. In addition, vitamin C protected rats form radiation-induced liver damage through activation of the antioxidant defense system including glutathione-s-transferase (GST), superoxide dismutase (SOD) and catalase (CAT) [128].

***Prebiotics*** Whilst oligosaccharides have not yet been tested for their radioprotective applications in preclinical settings, several flavonoids have been identified as potent radioprotectants [129,130]. For instance, radioprotective capacities of bioflavonoid propolis were seen in mice exposed to 9 Gy whole-body irradiation. This effect was confirmed by diminished primary DNA damage in leukocytes as well as delayed onset of mortality [131,132]. 

Interestingly, prebiotics can be delivered locally by probiotic bacteria. For instance, it was suggested that *L. reuteri* strain 121 produces prebiotic FOS and is therefore able to stimulate beneficial intestinal microbiota [133]. In addition, increased fecal abundance of butyrate-producing bacteria was reported to be associated with increased resistance against respiratory viral infections [134]. Alternatively, cyanobacterium *Arthrospira platensis* products (Spirulina) are rich in vitamins A, B6, B9, E, and C as well as polyphenolic phycochemicals, and have been shown to protect murine bone marrow cells exposed to γ rays [135].

***Probiotics***Table 2 provides an overview of preclinical studies investigating the effects of probiotic supplementation on radiation-induced GI toxicities. Of interest, although many health benefits of probiotics appear to be strain-specific [136], little variation is seen in the types of bacteria applied as probiotics in radiation research, with predominant use of *Lactobacilli*. This implies that *Lactobacilli* could have some core properties, which mediate protection against radiation-induced GI complications. To illustrate, preventive effects towards radiation-induced GI toxicities were seen with Microflorana^®^-F, an active probiotic mixture composed of *L. acidophilus*, *L. helveticus,* and *Bifidobacterium* spp., which improved both overall survival as well as bacterial contamination, even after abdominal exposure to 20 Gy [137]. In addition, improvements of small intestinal morphology were observed with probiotic *L. acidophilus* (strain not mentioned) supplementation in rats locally irradiated with ≤15 Gy [138]. Furthermore, reduced epithelial apoptosis and improved crypt survival was demonstrated upon probiotic *L. rhamnosus* GG ATCC 53103 supplementation without causing drastic shifts in bacterial compositions, following 12 Gy whole-body γ radiation [139]. Even more interesting, these effects of *L. rhamnosus* GG ATCC 53103-mediated small intestinal radioprotection extended to fractionated dosing regimens [140]. Besides preventive applications, therapeutic effects of *L. delbrueckii* subspecies *bulgaricus* B3 strain were observed in rats following a dose of 11 Gy directed to the abdominal-pelvic area [141,142]. Finally, *L. plantarum* 299v was shown to reduce GI injury and inflammation in rats exposed to 10 Gy lower abdominal radiation and even improved colonic anastomotic healing [143]. 

### 3.3. Clinical Evidence for Reduction of Radiation-Induced GI Toxicity

***Vitamins*** Due to preclinical success and overall safety, vitamins may be investigated for their radioprotective potential in clinical settings. A pilot study investigating vitamin E and C supplementation in prostatic or gynecologic cancer patients exposed to radiotherapy was shown to improve diarrhea and other symptoms, despite rectal pain. Furthermore, continuation of this supplementation up to 1 year resulted in sustained improvements of the symptoms [144]. To our knowledge, there have been no studies performed on the sole effect of vitamin A, B6, B9, or B12 in clinical settings of pelvic radiotherapy. 

***Prebiotics*** A recent, placebo-controlled clinical trial investigated oligosaccharide supplementation in patients with gynecological cancers who received radiotherapy with a cumulated dose of 52.2 Gy after surgery [145]. Although radiotherapy is well-reported to cause a decrease in fecal *Lactobacillus* and *Bifidobacterium* counts, administration of this prebiotic supplement was able to improve the recovery of both genera afterwards.

Although most of the data provide preclinical evidence, human clinical studies investigating polyphenolic phyco- and phytochemicals are scarce and often controversial, partly due to inter-individual variability in responsiveness.

***Probiotics***Table 3 summarizes evidence, which exists on probiotic applications in cancer patients exposed to radiotherapy, with a focus on the reduction of diarrhea and viral gastroenteritis [146]. Meanwhile, a meta-analysis and several randomized clinical trials have studied a plurality of probiotics and their therapeutic and preventive effects in radiotherapy-exposed patients [146]. Most of these trials primarily focused on radiotherapy-induced diarrhea and its effects on lower parts of the GI tract [147]. For instance, Delia et al. (2002, 2007) organized multiple clinical trials investigating a high-potency probiotic preparation VSL#3^®^ composed of four strains of *Lactobacilli* (*L. paracasei BP07, L. plantarum BP06, L. acidophilus BA05, and L. delbruekii subsp. bulgaricus BD08*), three strains of *Bifidobacteria* (*B. longum BL03, B. breve BB02, and B. infantis BI04*), and *Streptococcus salivarius subsp. thermophiles BT01*. Results suggested the effectiveness of VSL#3^®^ in preventing the occurrence of diarrhea in patients submitted to radiotherapy with direct and indirect improvements of their quality of life, as well as a good tolerance [148,149]. Other probiotics were also reported to have various beneficial effects on radiotherapy-induced diarrheic complications [150,151,152,153,154,155,156,157].

All this evidence as listed in Table 3 suggests that probiotics may have a beneficial role in prevention of radiation-induced diarrheic symptoms. However, most studies could only report modest effects, without affecting all diarrheic parameters including stool frequency, consistency, bowel movements, and the needs for anti-diarrheal medications simultaneously. In addition, no overall improvements in all study participants were reported suggesting inter-individual variability for probiotics’ success. Moreover, limited underlying modes of action or objective observations of protection against radiation-induced GI injuries have been described. Of note, the absence of serious adverse events reported in clinical trial reports should be interpreted with caution, especially since systemic infections associated with specific probiotic intake have been described elsewhere. For example, cases reporting sepsis or endocarditis and bacteremia following *L. rhamnosus* GG and *L. casei*, supplementation were recorded by Doron and Snydman (2015) [158]. *Bifidobacteria* are rarely associated with negative effects, yet bacterial sepsis and cholangitis were reported due to *Bacillus subtilis* intake [159]. Overall predisposing risk factors include immunosuppression, prior hospitalization, severe underlying comorbidities, previous antibiotic therapy, prior surgical interventions, critically ill patient status, and central venous catheters. Another study showed an increased risk of mortality associated with enteral administration of a multispecies probiotic preparation to prevent infectious complications in patients with predicted severe acute pancreatitis [160]. Therefore, cautious intake of probiotics is encouraged, especially in critically ill patients or in those at risk of non-occlusive mesenteric ischemia. 

### 3.4. Mechanisms of Radioprotection

There is only limited knowledge on the underlying mechanisms by which food supplements elicit their radio-protective effects. In the case of probiotics, general modes of action for *Lactobacillus* have been reviewed by Lebeer et al. [136,161]. In summary, its probiotic application assumes that the mechanisms underlying health-promoting capacities include pathogen inhibition and restoration of microbial homeostasis through microbe-microbe interactions, enhancement of epithelial barrier function, and modulation of immune responses. However, given the complexity of these three main functions, more experimental work is still required. 

#### 3.4.1. Antimicrobial Capacities

***Vitamins*** Interestingly, vitamin A has been associated with antiviral efficacy towards murine norovirus, acting through a significant increase in relative abundance of *Lactobacillus* sp. [162,163]. In addition, vitamin B6 was shown to eliminate *Salmonella Typhimurium* from the gut [164]. However, to our knowledge, antimicrobial actions for vitamins E, C, B9, and B12 have not yet been reported.

***Prebiotics*** Although prebiotics are—according to their definition—selectively used by host microorganisms to confer a health benefit, no direct antimicrobial actions have been reported. However, prebiotic research showed stimulated mucus production by Goblet cells and competitive interference with enteropathogens [165,166,167,168]. 

***Probiotics*** Probiotics were suggested to beneficially affect the host through direct effects on microbiota. Traditionally, probiotics—like commensal bacteria—can exert anti-pathogenic activities through nutrient competition, production of antimicrobials and/or competitive exclusion. For instance, metabolic cross-feeding is extremely interesting when *Lactobacilli*-produced lactic acid is converted into butyric acid by butyrate-producing colonic bacteria since butyrate exerts intestinal health-promoting capacities [169,170,171,172,173,174]. In addition to the general antimicrobial metabolite lactic acid, many probiotics may also produce bacteriocins, which are strain-specific peptides with direct antimicrobial activity either through membrane permeabilization, pH lowering, and/or capture of elements essential for microbial growth [175,176,177,178,179,180,181,182]. Furthermore, some *L. reuteri* strains produce additional antimicrobial reuterin [183]. Recently, oral administration of probiotic *L. casei* CRL 431 and *L. paracasei* CNCM I-1518 was described to increase Paneth cells as well as intestinal antimicrobial activity [184]. As Paneth cells contribute to small intestinal remodeling following whole-body γ irradiation, they should be explored in further detail for probiotic-induced radio-protective evidence [185]. Interestingly, antibacterial activity of probiotic *L. rhamnosus* GG and *L. acidophilus* LA towards *Salmonella Typhimurium* C5 was observed, both in vitro and in germ-free mice, without affecting the normal gut microbiota [186,187]. Besides nutrient competition, probiotics may compete for binding to host mucosa and thereby exclude intestinal pathogens and thus prevent bacterial translocation [188]. Intermicrobial binding or co-aggregation is thought to be another possible mechanism by which clearance of pathogens during mucus flushing is enhanced [189,190]. Whether these intermicrobial interactions by probiotics are able to reduce infections in vivo, remains to be largely determined. 

#### 3.4.2. Barrier-Enhancing Capacities

***Vitamins*** Antioxidative capacities of vitamin C were reported to significantly reduce the complaints of breast cancer patients one year post-surgery [191]. In parallel, antioxidative actions of vitamin E as well as vitamins B6 and B9/B12 were shown by reduced oxidative stress when rats’ intestinal barrier was subjected to different stressors [64,123,192]. 

Barrier protection by vitamin A was proposed by enhanced mucin synthesis [67,168]. In addition, vitamin A might regulate and maintain the intestinal epithelial barrier through IL-22 [193]. Specifically, retinoic acid, the most biological form of vitamin A, was reported to promote tolerance and reduce inflammation in rodent colitis models [194,195]. Other studies have described vitamin A as a stimulating agent for migration and proliferation of IECs whilst balancing the gut microbiome [193,196]. 

A combined cocktail of vitamins A, C, and E before irradiation was reported to reduce oxidative stress in small intestinal crypt cells, which then protected absorptive transport of sugars, amino acids, bile acids, and peptides [124,197]. Still, many gaps remain in our understanding of vitamin interactions with gut microbiota, mucosal immunity, and intestinal barriers, which are today’s research subjects.

***Prebiotics*** Similar to vitamins, prebiotics are studied for their antioxidant capacities to delay or prevent radiation-induced oxidative stress and consequent mucosal damage. Despite limited knowledge about prebiotic oligosaccharide antioxidant actions, FOS antioxidative and hepato-protective effects were observed in mice that were chronically exposed to oxidative stress mimicking accelerated ageing [198]. Furthermore, supplementation of FOS to constipated nursing-home elderly residents reduced lipid peroxidation levels, likely mediated by colonic bacteria in humans [199]. On the contrary, a large number of phytochemicals were reported to possess antioxidant properties by averting the continuation of the free radical chain reaction upon contact [200]. In humans, a phytonutrient mix tested as an ageing defense mechanism in UV radiation-induced skin damage was evaluated positively as it provided antioxidant protection and cellular repair as well as modulated inflammation [201]. 

Although the role of prebiotic oligosaccharides in barrier enhancement through tight junctions is rather disputable [202,203,204], in vitro research could show induced transcription and assembly of tight junction proteins following incubation with the most abundant flavonoid quercetin [205,206]. Unfortunately, their applications as protectants of the intestinal mucosal barrier in radiation-induced GI toxicity have not yet been investigated.

***Probiotics*** A possible way by which probiotics may exert radioprotection is through intestinal barrier enhancing modalities. For instance, safe and natural antioxidant additives that delay or prevent radiation-induced oxidative stress and consequent damage are of high interest. Probiotic antioxidant actions can be obtained through their metal ion chelating capacities [207,208]. Besides this, exopolysaccharides (EPS) of probiotic lactic acid bacteria have shown promising biological activities including antioxidant, antitumor, anti-biofilm formation by pathogens, and immunomodulation [209,210,211,212,213,214,215]. Furthermore, potential roles in stress resistance, adhesion, colonization, and host-bacteria interactions have been suggested [216,217,218]. Animal studies investigating the effects of feeding EPS-producing *Lactobacilli* demonstrated gut health benefits in part through altered gut microbiota composition and functioning, as well as favored antioxidant activities [219,220]. Another defense approach involves stimulation of the complex inherent antioxidant enzymatic systems, including SOD, CAT GST, glutathione peroxidase (GSH-Px) and glutathione reductase (GR), which are present in both the host and probiotic bacteria. Despite promising, preliminary results, therapeutic application of antioxidant enzymes is limited due to its short circulatory half-life, which restricts its bioavailability. Consequently, probiotics are now being exploited for their capacity of local antioxidant enzyme delivery. In the past, engineered *L. casei* BL23 strains producing SOD and CAT were shown to reduce or even prevent intestinal pathologies caused by ROS [221,222]. Recent comparative genome analysis suggests that also endogenous enzymatic activities could be used, with SOD being specific for *L. paracasei* strains and CAT for *L. casei* strain [223]. Similar beneficial observations of specific probiotic strains carrying antioxidant enzymes may also apply for cases of radiation-induced oxidative stress in the intestine. Alternatively, food supplements can also stimulate the host’s defense system through efficient activation of antioxidant actors, which was proven effective for a variety of probiotic bacteria tested in vivo [224,225,226,227,228,229,230]. On the other hand, probiotics can also produce diverse, non-enzymatic antioxidant metabolites such as glutathione (GSH), short chain fatty acids (SCFAs), and vitamin B9 [104,161]. This is highly interesting as radiation induces GSH depletion whilst maintaining ROS levels. This then increases the host’s susceptibility to oxidative tissue damage and hinders recovery of intestinal mucosa [231,232,233]. Unfortunately, it is difficult to increase circulating glutathione to a clinically beneficial amount through oral administration [234] and the applicability of GSH precursors (e.g., N- acetylcysteine) is limited by their toxic side effects [235,236]. To our knowledge, no studies in the context of intestinal radiation injury have been performed investigating probiotics that locally produce radio-protective GSH. Another feature shared by many probiotics is the ability of locally producing SCFAs of which butyrate has received particular attention for its beneficial effects on intestinal health [169,237]. For instance, butyrate was reported to stimulate a variety of colonic mucosal functions such as inhibition of inflammation and carcinogenesis, re-establishment of its defense barrier and decreasing oxidative stress whilst positively balancing gut microbiota [170,171,172,238]. For these reasons, lactate- and butyrate-producing bacteria are extremely interesting in the context of intestinal radiation injury, despite conflicting data obtained by randomized trials organized so far [173,174]. In addition, folate-producing *Bifidobacterium* strains tested in animal and human trials confirmed folate production and absorption upon intake, as well as enhanced fecal folate levels, without further investigations of their intestinal effects [239,240]. 

Radiation also negatively affects the expression of tight junctions-related proteins in an intestinal segment specific fashion contributing to the impairment of intestinal barrier integrity [26,241,242,243]. Both commensal bacteria and probiotics have been shown to enhance and restore intestinal barrier integrity in vitro and in vivo through tight junction regulation [244,245,246,247,248,249,250,251]. Specifically, probiotic *L. plantarum* WCFS1-mediated activation of TLR2 was reported to improve the intestinal barrier through changes in tight junction expression in human tissue [111]. A final intestinal barrier protection mechanism involves the mucus layer that is affected by external beam irradiation [252,253]. Adherence to IECs and mucosal surfaces has been suggested to be an important qualification for colonization of bacteria along the GI tract providing a competitive advantage in this ecosystem [161,254]. As such, probiotics can also minimize biofilm formation in which pathogens can aggregate [255]. Researchers have shown mucus production to be stimulated by Goblet cells as well as competitive interference with enteropathogens through the use of probiotics [256,257,258,259,260,261]. However, their applications as protectants of the intestinal mucosal barrier in radiation-induced GI toxicity have not yet been widely investigated. 

#### 3.4.3. Immunomodulatory Capacities

***Vitamins*** Although vitamins mainly exert radioprotective potential through antioxidant mechanisms, they have been shown to modulate the immune response, likely mediated by TLRs [194]. For instance, survival of 10 to 12 Gy whole-body irradiated mice was enhanced by vitamin E inhibiting pro-inflammatory cytokines IL-1β and -6, and reducing apoptosis in IEC [262]. In addition, vitamins B6 and B9 were shown to ameliorate the severity of murine colitis through reduction of *Salmonella Typhimurium* loads or augmentation of cellular methylation, respectively [263].

***Prebiotics*** Generally, little is known about immunomodulatory capacities of prebiotics in humans, and even less is reported in clinical radio-therapeutic settings. However, some preclinical studies showed possible immunosuppressive roles in cases of inflammatory bowel diseases [91,203,264]. The natural bioflavonoid propolis revealed successful, protective effects when mice were exposed to 9 Gy of γ rays, through an augmented activity of multiple immune cells [131,132]. 

***Probiotics*** Finally, current probiotic research is extremely interested in its ability to influence the immune response evoked by radiation. To this regard, probiotic interventions with *Lactobacilli* and/or *Bifidobacteria* have been shown to influence immune responses, likely mediated by TLRs [139,140,265,266,267,268,269]. Applications during radiotherapy showed, for example, that diarrhea could be prevented by live *L. acidophilus* plus *B. bifidum* (Infloran^®^), likely because of an improved immune status of the patients involved [152]. 

## 4. Discussion

With cancer survival rates improving over recent years, more patients are frequently suffering from treatment-induced side effects. For instance, cancer patients have reported GI symptoms due to pelvic radiotherapy, which induces injuries in healthy surrounding tissues. Unfortunately, our understanding of the biological complexities that underlie GI toxicity of healthy intestinal tissues initiated by pelvic radiotherapy is still limited. In all, there is still a long way ahead to provide patients with actual, effective prevention or treatment. 

Current advances in linking the pathobiology of radiation-induced GI symptoms with gut microbial dysbiosis have encouraged research on food supplements as promising actors for prevention and/or treatment of these symptoms. The scientific basis for studies applying dietary interventions in humans can be found in a large number of animal experiments that have identified potential pathophysiological mechanisms in the small and large intestine. For instance, interventional, preclinical trials have shown that some of these mechanisms can be blocked, leading to a significant reduction in radiation-induced GI damage. 

Given their safe character, food supplements like probiotics, prebiotics, and vitamins are very attractive to investigate for their potential in modulating the intestinal response to pelvic irradiation. In addition, research has shown evidence for their barrier enhancing, immunomodulatory and intermicrobial activity. Initial attempts in applying food supplements appear safe in the context of radiation-induced diarrhea, although the patient’s health status (e.g., immunocompromised patients) can affect this response. In addition to these negative complications of food supplement use, there is the issue of high inter-individual variability for its success. In future scenarios, patients may be screened to characterize their microbiome profile to subscribe the most appropriate food supplement, or a symbiotic combination, to the patient. 

Altogether, future research investigating the effects of radiation on the GI tract and the microbiome is warranted. First, it is important to stress that applied single fraction radiotherapy does not adequately reproduce the conditions of fractionated radiotherapy as used in patients. Therefore, the acute induced GI toxicity studied in experimental models may not fully represent clinical outcomes of patients. In addition, findings obtained from animal studies remain to be translated to diagnostic, prophylactic, or therapeutic treatments for humans. One caveat concerns a potential different radiosensitivity between animals and humans. Another hurdle is that microbiota members differ not only among host species but also between individual host organisms, and fluctuate in time. In addition, diet and different treatments (e.g., chemotherapy, surgery) are important confounding factors that differentiate and modulate the composition of the gut microbiota in an individual over time. Furthermore, it remains unclear whether an altered microbiome associated with a disease in humans causes, contributes to or merely is a consequence of the disease state. Hence, it will be a major challenge to explore strategies to translate the experimental data into a clinical therapy. To make further progress within the field of food supplement research, a number of basic issues still needs to be addressed, including optimal strain characterization together with quality control, dose optimization, clear definition of the desired effects, as well as (combined) formulation. Then, study endpoints—other than diarrhea—have to be defined accurately and a clinically meaningful study duration described. In parallel, regulatory aspects have to be clarified and appropriate guidelines for the evaluation of food supplements, whether as food or drug, need to be developed. Although the road of developing a good food supplement for radiotherapy patients seems long, promising evidence gathered from (pre)clinical studies should be a scientist’s driving force. 

## Figures and Tables

**Table 1:** Overview of experimental studies on probiotic strategies for improving general gastrointestinal functioning.

Probiotic	Gut Improving Effect	Type of Study	References
*Individual L. acidophilus* strains ADH or N2, or *L. bulgaricus (strain not mentioned)*, or *Streptococcus thermophiles (strain not mentioned)*	Survival in human stomach and adhesion to IECs ^†^	In vitro In vivo (healthy pigs and humans)	[98]
*L. rhamnosus* GG ATCC 53103	Prevention of cytokine-induced apoptosis in IECs ^†^	In vitro	[99]
	Prevention of *E. coli*-induced changes in epithelial barrier function	In vitro	[100]
	Restoration of intestinal integrity of murine ileum through occludin expression	In vivo (mice with alcoholic liver disease)	[101]
	Prevention of increased intestinal paracellular permeability in Caco-2 cells; Restoration of tight junction proteins such as ZO-1 ^†^, claudin-1, and occludin	In vitro	[102]
	Induction of inflammatory tolerance of the intestinal mucosa	In vitro In vivo (healthy mice)	[103,104]
	Local dampening of innate immune responses with desensitization towards luminal antigens	In vitro	[105]
	Effective treatment of acute gastroenteritis	In vivo (children with acute gastroenteritis)	[106]
	Faster recovery of acute non-bloody diarrhea	In vivo (children with acute diarrhea)	[107,108,109]
*B. bifidum* G9-1	Induction of mucosal protective factors including MUC2-4 ^†^, TGFβ1 ^†^ and TFF3 ^†^; Alleviation of diarrhea partly through protection against human rotavirus induced lesions	In vivo (mice pups with rotavirus gastroenteritis)	[110]
*L. plantarum* WCFS1	Restoration of tight junction proteins ZO-1 ^†^ and occludin	In vitro In vivo (healthy humans)	[111]
*L. reuteri* I5007	Restoration of tight junction proteins claudin-1, occluding, and ZO-1 ^†^	In vitro In vivo (healthy piglets)	[112]
*L. casei* DN-114 001	Prevention of transcription of numerous pro-inflammatory genes encoding cytokines, chemokines and adherence molecules	In vitro	[113]
	Restoration of tight junction protein ZO-1 ^†^ in Caco-2 cells	In vitro	[114]
Ultrabiotique^®^ (*L. acidophilus + B. lactis + L. plantarum + B. breve*)	Improvement of clinical symptoms and histological alterations; Down regulation of nitric oxide production by peritoneal macrophages; Enhancement of mucus production with modification of microflora	In vivo (mice with colitis)	[115]
*L. acidophilus* (strain not mentioned)	Overall attenuation of the severity of DSS ^†^-induced colitis, specifically by suppressing pro-inflammatory cytokines	In vitro In vivo (mice with colitis)	[116]
Mixture of *Streptococcus thermophilus + L. acidophilus + L. bulgaricus* (strains not mentioned)	No improvement of acute diarrhea	In vivo (children with acute diarrhea)	[117]

^†^ IEC = intestinal epithelial cell; ZO-1 = zonula occludens-1; MUC2 = mucin 2; TGFβ1 = transforming growth factor 1; TFF3 = trefoil factor 3; DSS = dextran sulfate sodium.

**Table 2:** Overview of preclinical studies on probiotic strategies for improving radiation-induced GI toxicity in tumor-free rodents.

Probiotic	Radiation Dose	Method of Supplementation	Duration of Supplementation	Results of Supplementation	References
Microflorana^®^-F *(L. acidophilus* + *L. helveticus* + *Bifidobacterium* spp)	Abdominal X irradiation with 1 × 20 Gy	Oral gavage of 1 mL of probiotic solution three times daily	Started seven days before the irradiation procedure and maintained until 14 days thereafter	Improved overall survival; Improved endotoxin levels; Reduced incidence of bacterial contamination	[137]
*L. acidophilus* (strain not mentioned)	Abdominal-pelvic irradiation with either 1 × 10, 15 or 20 Gy	Oral gavage of 2 mL of probiotic solution (10^8^ CFU ^†^)	Started six days before irradiation and maintained until three days thereafter	Improved morphology of the small intestine after 10 or 15 Gy; No improvements of jejunal villi height when irradiated with 15 Gy; No improvements when irradiated with 20 Gy	[138]
*L. rhamnosus* GG ATCC 53103	Whole body γ irradiation with 1 × 12 Gy	Oral gavage of probiotic solution (5 × 10⁷ CFU ^†^), daily	Three consecutive days before irradiation	Reduced epithelial apoptosis particularly at crypt bases; Improved crypt survival; No detectable shifts in bacterial compositions	[139]
		Oral gavage or intraperitoneal injection of lipoteichoic acid (5 mg/kg), a radioprotective agent in *L. rhamnosus* GG, daily	Three consecutive days before irradiation	Improved small intestinal crypt survival	[140]
	Total abdominal X irradiation with 7 or 8 × 4 Gy	Intraperitoneal injection of lipoteichoic acid (5 mg/kg), a radioprotective agent in *L. rhamnosus* GG, daily	One hour before each fractionated radiation dose	Improved post-radiation weight recovery and survival	
*L. delbrueckii* subsp. *Bulgaricus* B3 strain	Abdominal-pelvic γ irradiation with 1 × 11 Gy	Oral gavage of 2 mL of probiotic solution (10^10^ CFU ^†^/mL), daily	Seven consecutive days after irradiation	Reduced scores for inflammation and vascularity; Accelerated healing; Decreased bacterial translocation; Reduced diarrhea	[141,142]
*L. plantarum* 299v	Lower abdominal X irradiation with 2 × 10 Gy	Oral gavage of probiotic solution (2 × 10^9^ CFU ^†^), twice daily	Started one day after irradiation and was continued throughout the experiment for a maximum of 15 days, except for the operation day	Increased collagen content; Decreased mucosal myeloperoxidase activity	[143]

^†^ CFU = colony-forming units.

**Table 3:** Overview of clinical studies on probiotic strategies for treating radiation induced gastrointestinal toxicity in cancer patients.

Probiotic	Summary of Study	Method of Supplementation	Duration of Supplementation	Results	References
*L. acidophilus* NCDO 1748	Patients (*n* = 24) with gynecological tumors, 80 Gy	A formulated drink (≥2 × 10^9^ CFU ^†^) and 6.5% lactulose as bacterial substrate, once daily	Started five days prior to radiotherapy, daily throughout the radiotherapy period including the interval, and continued for 10 days thereafter	Reduced diarrhea	[150]
*L. casei* DN-114 001	Patients (*n* = 85) with gynecological tumors, 45–50 Gy with weekly cisplatin treatment (40 mg/m^2^)	A formulated drink (10^8^ CFU ^†^/g), three times daily	Started one week prior to radiotherapy	Improved stool consistency; No significant differences in need for rescue anti-diarrheal therapy, neither diarrhea severity	[156]
Infloran^®^ *(L. acidophilus and* *B. bifidum)*	Patients (*n* = 63) with locally advanced cervical cancer receiving 50 Gy with additional brachytherapy of four times 7 Gy with weekly cisplatin during radiotherapy procedure	Two oral capsules (2 × 10^9^ CFU ^†^/g of each bacteria), twice daily	Started 7 days prior to radiotherapy and maintained during radiotherapy	Reduced severity of diarrhea;Reduced need of rescue anti-diarrheal therapy; Improved stool consistency	[152]
VSL#3^®^ (*Streptococcus thermophilus* BT01, *B. breve* BB02, *B. longum* BL03, *B. infantis* BI04, *L. acidophilus* BA05, *L. plantarum* BP06, *L. paracasei* BP07, *L. delbrueckii subsp. bulgaricus* BD08)	Patients (*n* = 190) with pelvic tumors, 60–70 Gy	A formulation (450 × 10^9^ CFU ^†^/g), three times daily	Started on the first day of radiotherapy, for 6 to 7 consecutive weeks of therapy	Reduced number of patients suffering from radiation induced toxicity; Reduced severity of toxicity	[148,149]
	Patients (*n* = 239) with postoperative cervical, sigmoid or rectal tumors, 60–70 Gy			Improved number of bowel movements; Delayed need for additional anti-diarrheal therapy	
Bifilact^®^ (*L. acidophilus* LAC-361 and *B. longum* BB-536)	Patients (*n* = 246) with rectal, cervical, endometrial or prostatic cancers that had radiotherapy with or without surgery or chemotherapy	Oral capsules (1.3 × 10^12^ CFU ^†^), twice daily or three times daily	Started on the first day of radiotherapy and maintained up until the last day of radiotherapy	Reduced severity of diarrhea with standard dosing	[154,155]
“5” Strain Dophilus^®^ (*L. rhamnosus* HA-111, *B. Breve HA-129, L. acidophilus* HA-122, *B. longum* HA-135, *L. Casei* HA-108)	Patients (*n* = 42) with abdominal-pelvic cancers who received post-operative radiotherapy or radiotherapy with chemotherapy, 50–67 Gy	Oral capsules (6 × 10^12^ CFU ^†^), twice daily	Started on the first day of radiotherapy and maintained up until the last day of radiotherapy	Reduced incidence and severity of diarrhea	[157]
Antibiophilus^®^ (*L. rhamnosus* LCR 35)	Patients (*n* = 206) with several lower abdominal and pelvic tumors, 50 Gy	Oral capsules (1.5 × 10^9^ CFU ^†^), plus lactulose as bacterial substrate, three times daily	Started in case of diarrhea and maintained up to one week, depending on the response of the diarrhea	Improved number of bowel movements; Improved fecal consistency;	[151]
Gefiluss^®^ (*L. rhamnosus* GG ATCC 53103)	Patients (*n* = 39) with colorectal cancers receiving 45–50.4 Gy and 24 weeks of 5-FU chemotherapy	Oral capsules (1–2 × 10^10^ CFU ^†^), twice daily	Started at the start of adjuvant 5-FU chemotherapy and maintained for 24 weeks	Reduced severity of diarrhea; Less abdominal discomfort reported; Lower need for hospital care; Reduced need for chemotherapy dose adjustments due to bowel toxicity	[153]

^†^ CFU = colony-forming units.
